# Malaria prevalence and associated factors among symptomatic children aged under five years attending Sheko District Health Center, Southwest Ethiopia: A cross-sectional study

**DOI:** 10.1371/journal.pone.0295237

**Published:** 2023-12-01

**Authors:** Tadesse Duguma, Dessalew Wudineh, Aberash Assefa, Nebeyi Fisseha, Dassalegn Muleta

**Affiliations:** 1 Department of Medical Laboratory Science, College of Health Science and Medicine, Mizan-Tepi University, Mizan-Aman, Ethiopia; 2 Department of Pharmacology and Toxicology, School of Pharmacy, College of Medicine and Health Sciences, Mizan-Tepi University, Mizan-Aman, Ethiopia; Hawassa University College of Medicine and Health Sciences, ETHIOPIA

## Abstract

**Background:**

Malaria is a major cause of morbidity and mortality in children under the age of five worldwide. Although various malaria elimination measures have been implemented over the past decades, malaria remains a serious threat to public health, especially in tropical and subtropical areas. Ethiopia has set targets for eliminating malaria by 2030. No research has been conducted in the study area concerning malaria among children, who are the most malaria-prone segment of a community. The purpose of this study was to assess malaria prevalence and the factors associated with it among children under five years of age who attended the Sheko Health Center, Southwest Ethiopia, from June 1 to October 30, 2022.

**Materials and methods:**

An institutional-based cross-sectional study was employed from June 1 to October 30, 2022, at the Sheko Health Center. Capillary blood samples were collected from 286 randomly selected symptomatic children. Data on socio-demographics and associated factors were collected using a pre-tested structured questionnaire, and data on parents’ and guardians’ knowledge about malaria was recorded on Excel 2016 Spreadsheets after interviewing them, and their responses were presented by a frequency table. Data were entered into Epi Data Manager (v4.0.2.101) and analyzed using the Statistical Package for Social Sciences (SPSS) version 25. Associated factors of malaria were analyzed using bivariate and multivariable logistic regression, and statistical significance was set at P < 0.05.

**Result:**

Overall, 23.4% (95% CI = 18.6–28.8%) malaria infection was recorded among the children whose blood samples were examined, with *Plasmodium falciparum*, *Plasmodium vivax*, and mixed infections (both species) representing 52.2%, 34.3%, and 13.4% of the cases, respectively. The majority of the parents or guardians believed that malaria is transmissible but could be prevented, and 80% of them considered mosquito bites to be the main mode of malaria transmission. Insecticide-treated net (ITN) was mentioned as a malaria prevention strategy by more than half of the respondents, while indoor residual spraying (IRS) was considered only by 19.6%. Based on multivariable logistic regression analysis, a significant association was found in children between the ages of 12 and 36 months (adjusted odds ratio = 5.050; 95% CI: 1.964–12.982), children who lived in rural areas (adjusted odds ratio = 2.901; 95% CI: 1.439–5.845), and children who did not use ITN the past two weeks before sample collection (adjusted odds ratio = 3.341; 95% CI: 1.646–6.781).

**Conclusion:**

This study revealed a high malaria prevalence among children aged under five years. Attention must be paid to improving the coverage of the ITN and its use in the study area, which could help reduce the risk of mosquito bites. Health education for the guardians of the children could also help to raise awareness about the prevention and control strategies for malaria transmission and further reduce the impact of the disease.

## Introduction

Malaria is a major public health problem that still causes disease and death. It significantly contributes to poverty in areas with few resources, particularly in tropical regions around the world. Data from 85 countries with high malaria incidence show an increase in malaria cases between 2019 and 2020 [1]. However, the malaria mortality rate decreased by 62% from 2000 to 2019, from 148 to 56 cases per 100,000 people at risk, before rising to 60 in 2020 and again decreasing to 58 in 2021. According to the WHO African region report, deaths due to malaria decreased from 841,000 in 2000 to 541,000 in 2018, before increasing to 599,000 in 2020 and again decreasing to 593,000 in 2021 [2]. Based on the 2016 WHO report, malaria prevalence among children under the age of five years was 16% globally [3]. In the same year, Ethiopia had a prevalence of 0.6% [1]. Children under the age of five years, pregnant women, HIV/AIDS patients, non-immune migrants, mobile communities, and travelers have a higher risk of getting infected with malaria parasites and developing serious health complications [4, 5]. Children who experience severe malaria attacks can experience long-lasting neurological effects and mild and severe episodes that can cause developmental and cognitive problems [6, 7].

Malaria remains among the top 10 diseases in the district, as evidenced by the district health department report, which adds to the remoteness of the area with low infrastructure coverage, making the study area one of the hardest-to-reach areas in the region. Investigating the malaria prevalence in such an area is of paramount importance to the community of the study area, particularly children (vulnerable groups). Malaria affects children in different ways because children typically lack acquired immunity, i.e., they are more likely to experience severe malaria, also known as cerebral malaria, which can result in coma or seizures and quickly lead to death. Moreover, the side effects of recurrent infections like anemia raise the chance of death in the early months of life and result in low birth weight when they occur during pregnancy [8].

The national health sector transformation plan (HSTP) for Ethiopia’s four-year period of 2015/16 to 2019/20 was intended to be matched with the national malaria control strategy (NMSP) that was created by the Ethiopian government for the years 2017–2020. The suggested targets for the 2017–2020 NMSP include maintaining nearly zero malaria mortality (1 death per 100,000) by 2020, a 40% decrease in malaria cases by 2020, and the elimination of malaria in Ethiopia by 2030 [9, 10].

Previous studies, which were carried out in areas where malaria is endemic and is a primary cause of morbidity and mortality in children under five years of age in Ethiopia, only focused on the adult population [3]. There hasn’t been much research done on the epidemiological profile of malaria in children living in low-transmission settings [10]. This may be predisposed by the resource limitations of the country, making the Ministry of Health give due attention only to high malaria transmission settings (hot spots). This study aims to fill a major information gap by determining the prevalence and contributing factors of malaria infection among children under the age of five living in the study area. Moreover, the results of this study will aid clinical judgment and spark additional research that could help plan effective ways to prevent and control the disease and will play a role in meeting the goal of the country’s effort to eliminate malaria by 2030.

## Materials and methods

### Study design, setting, and period

An institutional-based cross-sectional study was conducted in the Sheko district at Sheko Health Center, southwest Ethiopia, from June 1 to October 30, 2022. The district’s boundaries are Debub Bench to the south, Guraferda to the west, Sheka Zone to the north, Gambela Region to the northwest, and Semien Bench to the east. The health center was established in 1961 and is located 588 km from Addis Ababa, Ethiopia, and 20 km from Mizan Teferi, which is one of the major cities of the Southern Nations, Nationalities, and People’s Region. There is a delivery department, an outpatient department, a laboratory department, an emergency room, an antenatal care unit (ANC), and a tuberculosis clinic at the health center. The health center has currently provided different health services for approximately 71,742 people (35,931 males and 35,811 females).

### Study participants

Children aged under 5 years who attended the Sheko district health center from June 1 to October 30, 2022, were enrolled, and the parents or guardians of the children were also considered participants for the face-to-face interview. Children aged under 5 years with malaria-related symptoms who visited the Sheko district health center during the study period and fulfilled the inclusion criteria were enrolled as study participants.

### Eligibility criteria

#### Inclusion and exclusion criteria

Children aged ≤ 59 months who had malaria symptoms [11] and were available for selection during the study period were included. The participants of the study were children below five years of age and were not capable of forming an opinion and assessing the information presented to them, which made it difficult for the investigators to obtain written assent for participation. So, after a brief explanation of the study’s objectives and procedures, verbal assent from the children and written informed consent from the parents or guardians were obtained before data collection. Children who were on antimalarial medication during the data collection period were excluded.

### Sample size and sampling technique

The sample size was determined by the following formula.

$$n=\frac{{\left(Z_{\frac{\alpha }{2}}\right)}^{2}p\left(1-p\right)}{d^{2}}$$

Where n = sample size, Zα/2 = 1.96, the standard normal variation at 95% confidence level, d = error margin, 0.05, p = prevalence probability of the prevalence of disease, 22.8% (0.228) [12], and q = failure probability, which is 1-p = 0.772, using the above formula, we have

$$\frac{1.96^{2\;}\times \;0.228\times (1-0.228)}{0.05^{2}}\;\approx \;270$$

After adding a 10% non-response rate the total sample size was calculated to be **297**.

### Sampling technique

Children who visited the health center laboratory for blood film examination and met eligibility criteria were recruited by a systematic random sampling technique, considering the flow of cases in the health center the previous year. The sampling frame was obtained from the registered information from the health facilities in the area studied, and accordingly, there were approximately 1414 children aged under 5 years who received services such as immunization or vaccination at health institutions in the catchment. The kth interval was calculated as (4.76 ≈ 5). Therefore, the study participants who met the inclusion criteria were picked every fifth time after randomly selecting the first child using the lottery system. Finally, 297 children who met the inclusion criteria were included in the study.

#### Dependent variable

Malaria prevalence.

### Independent variables

Some of the independent variables were age, sex, residence, educational status, occupational status, family monthly income, insecticide-treated net (ITN) use, mosquito/vector breeding site, insecticidal residual spraying (IRS), and family size…

### Data sources/measurement

Face-to-face interviews with parents or guardians of study participants (children) were used to collect sociodemographic information and related factors. A 6 μL and 2 μL capillary blood sample was taken aseptically from the children’s fingers or big toes using sterile blood lancets to prepare thick and thin blood film smears. To reduce the occurrence of any possible bias, a systematic random sampling technique was used.

### Sample processing

Frosted slides were used to make a thin and thick blood smear, and the blood films were air-dried before the thin film was fixed with absolute methanol. Following the standard operating procedure, both thin and thick blood smears were stained for 10 minutes with the Giemsa staining working solution. The blood film slides were examined by experienced laboratory technicians at the Sheko District Health Center.

### Statistical methods

Data were entered into Epi Data Manager (v4.0.2.101) and analyzed using the Statistical Package for Social Sciences (SPSS) version 25. Descriptive statistics were used to summarize the demographic profile of the study participants. Bivariate and multivariable logistic regression analyses were performed, and a P-value of less than 0.05 was taken as statistical significance.

### Blood film microscopy

A thick and thin blood film was prepared, and a thin smear was fixed with absolute methanol in the field and transported to the Mizan-Tepi University parasitology laboratory. It was stained with a 10% working solution of Giemsa for 10 minutes. The slides were examined using the oil immersion objective of the microscope by two experienced lab technicians independently. A third technician, who was unaware of the preceding results, was tasked with resolving any discrepancies. The blood film was recorded as positive or negative after examining 200 high-power fields of the thick smear [13, 14].

### Malaria parasite density

The formula below was used to determine the number of malaria parasites per microliter (μL) of blood, assuming that the patient’s total leukocyte count was 8000/μL. The parasite count multiplied by 8000 WBC/ μl of blood is the parasite burden. Consequently, the malaria parasite density was classified as high parasitemia with a parasite density of (≥ 5,000 parasites/μL of blood), moderate with a parasite density of (1000 to 4,999 parasites/μL of blood), and low with a parasite density of (< 1000 parasites/μL of blood) [15]. The number of parasites (asexual)/μL of blood (thick film):

$$\text{Parasites}/\mu \text{L}=\frac{\text{parasite}\;\text{counted}}{\text{WBC}\;\text{counted}}\times \text{WBC}/\mu \text{L}$$

If the number of WBCs is unknown, it can be assumed to be 8000/μL.

### Operational definitions

Symptomatic children: refers to children who had malaria-related symptoms (fever, i.e., axillary temperature ≥ 37.5°C, chills, headache, vomiting, joint pain) within the past 2 days and at the time of examination and the presence of malaria parasites in their blood [16, 17].

### Data quality control

In the test methods, a checklist, a structured questionnaire, and standard operating procedures (SOP) for malaria microscopy were employed to ensure the quality of the data. Giemsa’s working solution was prepared by filtering the crystals using Whatman filter paper. Additionally, the glass slides were labeled in such a way that the slide code matched the particular child’s file. A sterile automatic lancet was used for each child to draw the blood sample, and a microscopic examination of the blood films was performed by three experienced laboratory technicians.

### Ethics statement

Ethical clearance with approval number CHS/00982/22 was obtained from the College of Health Science and Medicine at Mizan-Tepi University. The objective of the study and the procedure were explained to the study participants before blood sample collection. Verbal assent from the children and written informed consent from the parents or guardians were obtained before data collection.

## Result

### Sociodemographic characteristics of the study participants

A total of 286 children aged under 5 years with malaria symptoms participated in the study, and a response rate of 96.3% was recorded from their parents or guardians. Of the children, 23.4% (95% CI = 18.6–28.8) were confirmed to be positive for *Plasmodium* species. More than 60% of the study participants were urban dwellers; more than one-third of the study participants were in the age range of 12–59 months; and more than half of them were male [Table 1].

**Table 1:** Sociodemographic variables of children aged under five years at Sheko District Health Center, Southwest Ethiopia, 2022.

Variables	Categories	n (%)
Age (months)	< 12	85 (29.7)
	12–36	102 (35.4)
	37–59	99 (34.4)
Sex	Male	148 (51.7)
	Female	138 (48.3)
Residence	Urban	176 (61.5)
	Rural	110 (38.5)
Total		286 (100)

### Malaria prevalence

Children between the ages of 37 and 59 months and those between 12 and 36 months had a higher malaria prevalence compared to children under the age of 12 months. In comparison, males had a higher malaria prevalence than females. In terms of the distribution of *Plasmodium* species, *Plasmodium falciparum (P*. *falciparum)* was comparatively more prevalent in both sexes than *Plasmodium vivax (P*. *vivax)*. More than 60% of the study participants were urban dwellers, while the malaria parasite was more common among children living in rural areas [Table 2].

**Table 2:** Malaria prevalence among children aged under five years at Sheko District Health Center, Southwest Ethiopia, 2022.

Variables	Categories	n (%)	Positive	*P*. *falciparum*	*P*. *vivax*	Mixed	Negative n (%)
			n (%)	n (%)	n (%)	n (%)	
Age (months)	< 12	85 (29.7)	9 (3.1)	5 (1.7)	3 (1.0)	1 (0.3)	76 (26.6)
	12–36	102 (35.4)	25 (8.7)	14 (4.9)	9 (3.1)	2 (0.7)	77 (26.9)
	37–59	99 (34.4)	33 (11.5)	16 (5.6)	11(3.8)	6 (2.1)	66 (23.1)
Sex	Male	148 (51.7)	39 (13.6)	19 (6.6)	14 (4.9)	6 (2.1)	109 (38.1)
	Female	138 (48.3)	28 (9.8)	16 (5.6)	9 (3.1)	3 (1.0)	110 (38.5)
Residence	Urban	176 (61.5)	24 (8.4)	12 (4.2)	9 (3.1)	3 (1.0)	152 (53.2)
	Rural	110 (38.5)	43 (15.0)	23 (8.0)	14 (4.9)	6 (2.1)	67 (23.4)
Total		286 (100)	67 (23.4)	35 (12.2)	23 (8.0)	9 (3.1)	219 (76.6)

### Malaria parasite density

Moderate parasitemia predominated in terms of malaria parasite density, followed by low and high parasitemia. Nearly half of the children had parasite densities of (5000 parasites/μL of blood), 35.8% had densities of (1000_4,999 parasites/μL of blood), and 14.9% had densities of (1000 parasites/μL of blood). Male study participants made up 57.6% of the total and had a greater moderate parasitemia than female participants, although there was no statistically significant difference in parasite density (p = .066). The highest parasite density in moderate parasitemia was seen in the age range of 37 to 59 months, and parasitemia was statistically significant across all age ranges (p = .002). Additionally, there were statistical differences (p = .001) between residents of rural and urban areas, with 60.6% of those in rural areas having moderate parasitemia [Table 3].

**Table 3:** Density of malaria parasite by age, sex, and place of residence among children aged under five years attended at the Sheko District Health Center, Southwest Ethiopia, 2022.

Variable	Categories	Parasite density			Total (%)	P-value
		Low (%)	Moderate (%)	High (%)		
Age (months)	< 12	4 (6.0)	4 (6.0)	1 (1.5)	9 (13.4)	**0.002**
	12–36	9 (13.4)	14 (20.9)	2 (3.0)	25 (37.3)	
	37–59	11 (16.4)	15 (22.4)	7 (10.4)	33 (49.3)	
Sex	Male	17 (25.4)	19 (28.4)	3 (4.5)	39 (58.2)	0.066
	Female	7 (10.4)	14 (20.9)	7 (10.4)	28 (41.8)	
Residence	Urban	9 (13.4)	13 (19.4)	2 (3.0)	24 (35.8)	**< .001**
	Rural	15 (22.4)	20 (29.9)	8 (11.9)	43 (64.2)	
Total		24 (35.8)	33 (49.3)	10 (14.9)	67 (100)	

### *Plasmodium* species distribution

**Fig 1:**
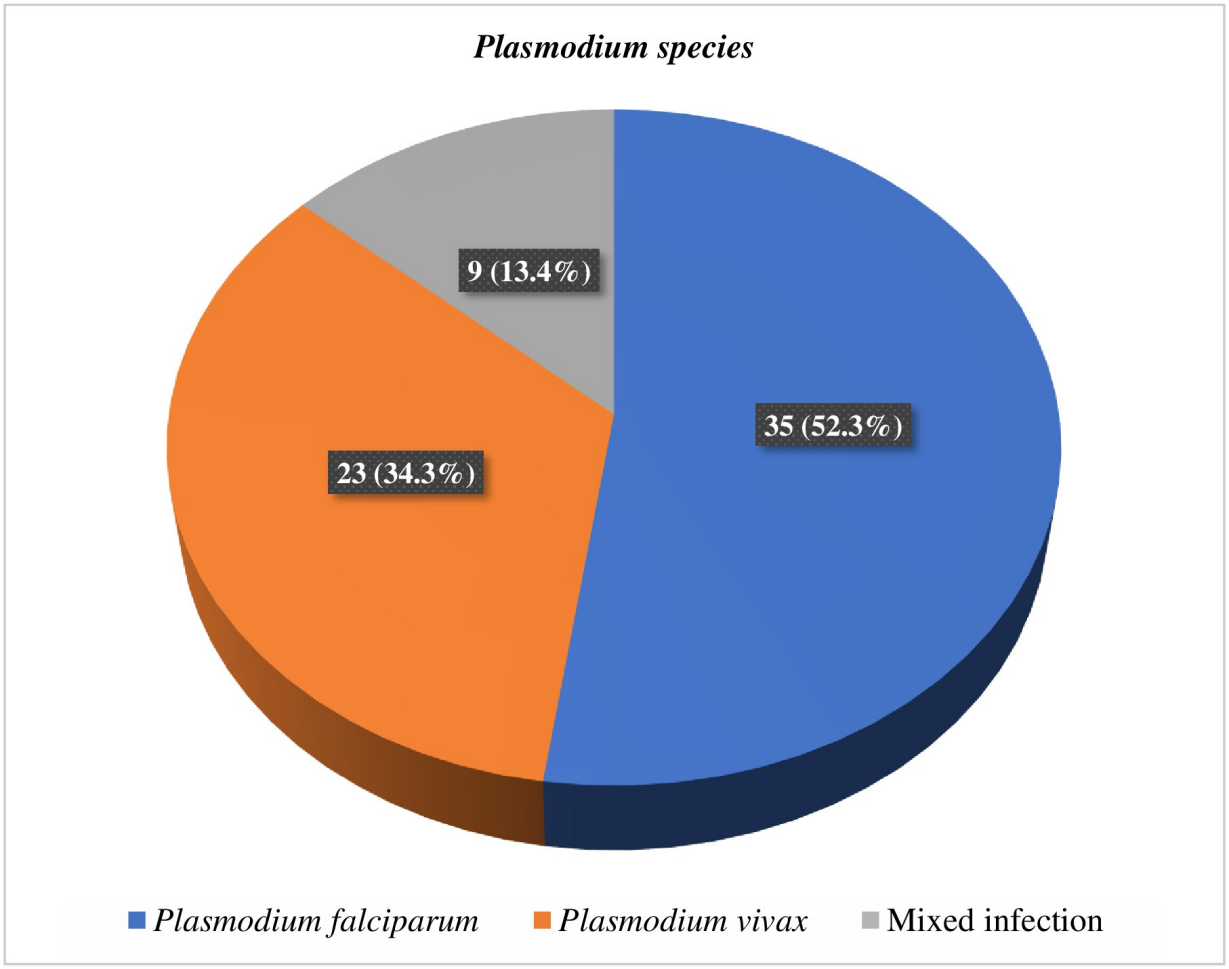
Distribution of *Plasmodium* species among children aged under five years attended at Sheko District Health Center, 2022.

The most common *Plasmodium* species in the country are *P*. *falciparum* and *P*. *vivax*. They accounted for 52.3% and 34.3% of the malaria prevalence recorded in this study, respectively, while the remaining 13.4% was contributed by a mixed infection of both species [Fig 1].

### Parent or guardian’s knowledge of malaria in the study area

The analysis of the parents’ or guardians’ knowledge showed that 94.1% had at least heard of malaria, the majority of the parents or guardians believed that malaria is transmissible but can be prevented, and 80% of them considered mosquito bites as the main mode of malaria transmission. Insecticide-treated nets were mentioned as a malaria prevention strategy by more than half of the respondents, while indoor residual spraying (IRS) was considered only by 19.6% [Table 4].

**Table 4:** Characteristics and knowledge of the parents or guardians about malaria in children aged under 5 years attended at the Sheko District Health Centre, Southwest Ethiopia, 2022.

Variables (Questions)	Participant response	Number n (%)
Educational Status	No formal education	88 (30.8)
	Primary (1–8)	62 (21.7)
	Secondary (9–10)	50 (17.5)
	Preparatory school (11–12)	56 (19.6)
	Diploma and above	30 (10.5)
Monthly income of the family (in Ethiopian Birr)	< 500	86 (30.1)
	500–1000	135 (47.2)
	1001–2000	44 (15.4)
	>2000	21 (7.3)
Family size	< 5 members	212 (74.1)
	≥ 5 members	74 (25.9)
Knowledge of malaria	Yes	269 (94.1)
	No	17 (5.9)
Source of information	Television	83 (29.0)
	Radio	62 (21.7)
	Health extension workers	96 (33.6)
	Awareness campaigns	45 (15.7)
Symptoms of Malaria	Fever	89 (31.1)
	Chills	41 (14.3)
	Vomiting	32 (11.2)
	Diarrhea	20 (7.0)
	Appetite loss	29 (10.1)
	Temperature > 37.5°C	35 (12.2)
	Others	40 (14.0)
Malaria can be treated	Yes	268 (93.7)
	No	18 (6.3)
Malaria can be transmitted from person to person	Yes	264 (92.3)
	No	22 (7.7)
Malaria is preventable	Yes	272 (95.1)
	No	14 (4.9)
Mode of malaria transmission	Bite of mosquitoes	229 (80.1)
	Contact with patients	14 (4.9)
	Unclean water	25 (8.7)
	Bad weather	18 (6.3)
Malaria prevention methods	ITN	156 (54.5)
	IRS	56 (19.6)
	Use of antimalarial drugs	45 (15.7)
	Environmental sanitation	29 (10.1)
Total		286 (100)

**ETB =** Ethiopian Birr, **ITN** = Insecticide treated net, **IRS** = insecticidal residual spray

### Prevalence of malaria and the associated factors

This study revealed that children who live close to a vector breeding site have a prevalence of sixty-five (22.7%) malaria infections, while children whose homes are not treated with insecticide chemicals have a high prevalence of 46 (16.1%) cases, and children whose homes have holes or cracks in the walls have a prevalence of fifty-three (18.5%). According to the findings of the study, households that had access to ITN were better protected than those that did not. Although the use of ITNs is the most important factor in malaria prevention, the number of ITNs available per family must also be considered. The more ITNs a household has, the lower its risk of developing malaria. Children who regularly slept on an ITN had a comparatively low prevalence of malaria [Table 5].

**Table 5:** Associated factors concerning the malaria burden among children aged under five years, attended at the Sheko District Health Center, Southwest Ethiopia, 2022.

Variable	Category	Malaria status		Total n (%)
		Positive n (%)	Negative n (%)	
Age (months)	< 12	9 (10.6)	76 (89.4)	85 (29.7)
	12–36	25 (24.5)	77 (75.5)	102 (35.7)
	37–59	33 (33.3)	66 (66.7)	99 (34.6)
Sex	Male	39 (26.4)	109 (73.6)	148 (51.7)
	Female	28 (20.3)	110 (79.7)	138 (48.3)
Residence	Urban	24 (13.6)	152 (86.4)	176 (61.5)
	Rural	43 (39.1)	67 (60.9)	110 (38.5)
Availability of ITN	Yes	26 (14.9)	149 (85.1)	175 (61.2)
	No	41 (36.9)	70 (63.1)	111 (38.8)
Number of ITN in the house	One ITN	15 (14.3)	90 (85.7)	105 (36.7)
	≥ 2 ITNs	11 (15.7)	59 (84.3)	70 (24.5)
ITN used the past two weeks before sample collection	Yes	21 (12.4)	148 (87.6)	169 (59.1)
	No	46 (39.3)	71 (60.7)	117 (40.9)
Frequency of ITN usage	Regularly	7 (7.3)	89 (92.72)	96 (33.6)
	Sometimes	19 (24.1)	60 (75.9)	79 (27.6)
House sprayed with IRS	Yes	21 (12.3)	150 (87.7)	171 (59.8)
	No	46 (40.0)	69 (60.0)	115 (40.2)
Frequency of IRS usage (per year)	Once	15 (18.8)	65 (81.2)	80 (28.0)
	≥ Twice	6 (6.6)	85 (93.4)	91 (31.8)
House has windows	Yes	57 (23.8)	182 (76.2)	239 (83.6)
	No	10 (21.3)	37 (78.7)	47 (16.4)
Windows have screen	Yes	21 (15.9)	111 (84.1)	132 (46.2)
	No	46 (29.9)	108 (70.1)	154 (53.8)
The wall of the house has a hole in it	Yes	53 (29.9)	124 (70.1)	177 (61.9)
	No	14 (12.8)	95 (87.2)	109 (38.1)
House has eaves	Yes	29 (17.7)	135 (82.3)	164 (57.3)
	No	38 (31.1)	84 (68.9)	122 (42.7)
Presence of mosquito breeding site near home	Yes	65 (52.8)	58 (47.2)	123 (43.0)
	No	2 (1.2)	161 (98.8)	163 (57.0)
Distance from the vector breeding site (Kilometer)	< 2 Km	42 (43.8)	54 (56.2)	96 (33.6)
	≥ 2 Km	25 (13.2)	165 (86.8)	190 (66.4)
Total		67 (23.4)	219 (76.6)	286 (100)

**ITN** = Insecticide treated net, **IRS** = insecticidal residual spray, **Km** = Kilometer

### Associated factors for malaria infection

More than 60% of the study participants reported having access to ITN, and 59.8% of them had used IRS services in the previous year. More than half of the guardians responded that their children used an ITN during the past two weeks before blood sample collection, while only 33.6% of the children slept under an ITN regularly over the previous two weeks. The multivariable logistic regression model was fitted considering variables from binary logistic regression with P-values of ≤ 0.25. Age (12 to 36 months), residence, child who did not use ITN the past two weeks before sample collection, frequency of ITN use, and presence of vector breeding sites close to the homes of children were selected as candidates for analysis. In the multivariable logistic regression analysis, children in the 12-36-month age interval were found to be more likely to be infected with malaria (AOR = 5.050; 95% CI: 1.964–12.982) than those under 12 months and older than 3 years; similarly, children who were rural residents were more prone to malaria infection than their counterparts (AOR = 2.901; 95% CI: 1.439–5.845). Furthermore, children who did not use ITN for the past two weeks before sample collection had a 3.3 times greater chance of contracting malaria than children who did not use it (AOR = 3.341; 95% CI: 1.646–6.781). Furthermore, children who used ITN only occasionally or infrequently had a higher risk of contracting malaria than those who used ITN regularly (AOR = 4.375; 95% CI: 1.709–11.203), and children who lived near a vector breeding site had a higher risk of infection (AOR = 33.782; 95% CI: 8.668–131.659) than those who lived far away from a vector breeding site [Table 6].

**Table 6:** Analysis of bivariate and multivariable logistic regression for the associated factors of malaria in children aged under five years who attended the Sheko District Health Center, southwest Ethiopia, from June 1 to December 30, 2022.

Variables	Category	Malaria		Odds ratio			
		Positive n (%)	Negative n (%)	COR (95% CI)	P-value	AOR (95% CI)	P-value
Age (months)	< 12	9 (10.6)	76(89.4)	Ref		Ref	
	12–36	25 (24.5)	77 (75.5)	0.365 (0.160,0.832)	0.017	5.050 (1.964, 12.982)	**0.001**
	37–59	33 (33.3)	66 (66.7)	0.237 (0.106,0.531)	0.001	1.599 (0.749, 3.417)	0.225
Sex	Male	39 (26.4)	109 (73.6)	1.406 (0.809,2.444)	0.228		
	Female	28 (20.3)	110 (79.7)	Ref		Ref	
Residence	Urban	24 (13.6)	152 (86.4)	Ref		Ref	
	Rural	43 (39.1)	67 (60.9)	0.246 (0.138,0.438)	0.001	2.901 (1.439, 5.845)	**0.003**
Availability of ITN	Yes	26 (14.9)	149 (85.1)	Ref		Ref	
	No	41 (36.9)	70 (63.1)	0.298 (0.169,0.526)	0.001	1.880 (0.919, 3.848)	0.084
Number of ITN in the house	One ITN	15 (14.3)	90 (85.7)	0.737 (0.261,2.079)	0.564		
	≥ 2 ITNs	11 (15.7)	59 (84.3)	Ref		Ref	
ITN used the past two weeks before sample collection	Yes	21 (12.4)	148 (87.6)	Ref		Ref	
	No	46 (39.3)	71 (60.7)	0.219 (0.122,0.395)	0.001	3.341 (1.646, 6.781)	**0.001**
Frequency of ITN usage	Regularly	7 (7.3)	89 (92.72)	Ref		Ref	
	Sometimes	19 (24.1)	60 (75.9)	0.082 (0.041,0.164)	0.001	4.375 (1.709, 11.203)	**0.002**
House sprayed with IRS	Yes	21 (12.3)	150 (87.7)	Ref		Ref	
	No	46 (40.0)	69 (60.0)	0.873 (0.492,1.550)	0.644		
Frequency of IRS usage	Once a year	15 (18.8)	65 (81.2)	0.455 (0.261,0.794)	0.006	0.498 (0.247, 1.004)	0.051
	≥ Twice a year	6 (6.6)	85 (93.4)	Ref		Ref	
House has windows	Yes	57 (23.8)	182 (76.2)	1.159 (0.542,2.476)	0.704		
	No	10 (21.3)	37 (78.7)	Ref		Ref	
Windows have screen	Yes	21 (15.9)	111 (84.1)	Ref		Ref	
	No	46 (29.9)	108 (70.1)	0.444 (0.249,0.793)	0.006	1.746 (0.871, 3.501)	0.116
The wall of the house has a hole	Yes	53 (29.9)	124 (70.1)	0.345 (0.181,0.658)	0.001	0.997 (0.484, 2.057)	0.994
	No	14 (12.8)	95 (87.2)	Ref		Ref	
House has eaves	Yes	29 (17.7)	135 (82.3)	Ref		Ref	
	No	38 (31.1)	84 (68.9)	0.475 (0.273,0.827)	0.009	1.319 (0.666, 2.614)	0.427
Vector breeding site near home	Present	65 (52.8)	58 (47.2)	0.011 (0.003,0.047)	0.001	33.782 (8.668,131.659)	**0.001**
	Absent	2 (1.2)	161 (98.8)	Ref		Ref	
Distance from vector breeding site (Kilometer)	< 2 Km	42 (43.8)	54 (56.2)	0.195 (0.109,0.349)	0.001	1.170 (0.544, 2.516)	0.687
	≥ 2 Km	25 (13.2)	165 (86.8)	Ref		Ref	
Total		67 (23.4)	219 (76.6)				

**Note: The P-value <0.05** was taken as statistically significant, **Ref** represents the reference category in both bivariate and multivariate logistic regression analysis, **COR** = crude odds ratio, **AOR** = adjusted odds ratio, **CI** = confidence interval, **ITN** = Insecticide-treated net, **IRS** = insecticidal residual spray

## Discussion

Malaria remains a public health issue for children aged under five years in the study area. The overall prevalence of malaria was 24.3% (95% CI = 18.6–28.8) among children who visited the Sheko district health center, which is comparable to the one obtained from southern Ethiopia (22.1%, 22.8) [5, 9], Ethiopia (22.03%, 24.6%) [18, 19], and Nigeria (22.6%) [20]. The malaria prevalence recorded in this study was higher compared to that found in a study conducted in Nigeria (10.3%) [21], Northwest Ethiopia (8.7%, 14.7%) [22, 23], Ethiopia (3.9%) [24], Madagascar (7.8%) [25], sub-Saharan Africa (18.8%) [26], and Uganda (19.5%) [27]. On the contrary, the prevalence in the current study area was lower compared to another study from Ethiopia (64%) [28], Burkina Faso (49.7%) [29], and Ghana (43.0%) [30]. This heterogeneity can also be the result of geographical variances and malaria prevention and control programs implemented in study areas. The two species of *Plasmodium* that were identified in the blood of children represented 52.3% and 34.3% of infections, respectively. This result is consistent with Ethiopia’s national *Plasmodium* species distribution [31–33]. According to this study, as children get older, it becomes less common for them to have a *Plasmodium* infection. This could be a result of the fact that these children reside in regions where malaria transmission is stable and can build age-related protective immunity from repeated exposure to infected mosquito bites [34].

Most of the malaria-positive children had moderate parasitemia, followed by low and high parasitemia levels, respectively, making up 49.3%, 35.8%, and 14.9% of the infected children. This is concurrent with previous studies conducted in northern Ethiopia [23], and in contrast, a study in Tanzania showed a high parasite density [35]. Various studies in Ethiopia [24, 28] have shown that the density of the malaria vector and living close to a body of water, such as a river or stream, could be important factors that influence malaria transmission. Although parents or guardians of the majority of children who participated in the study had a better understanding of the disease malaria, children of those families who reside in rural settings were found to have a higher malaria prevalence compared to their counterparts. A bite of mosquitoes was mentioned as a malaria mode of transmission by the parents or guardians of more than three-fourths of the children who participated in this study. Insecticide-treated nets (ITNs) were mentioned as a malaria prevention strategy by more than half of the respondents. More than sixty percent of the guardians of the children reported they had at least one ITN per household, which is higher compared to the ITN availability reported by a study conducted by Zerdo et al. (19.3%) [36]. More than eighty percent of the respondents in this study associated the mosquito bite with contracting malaria, which is better compared to the one recorded in a study from West Ethiopia [37], with 70% of the caregivers replying that mosquito bites contribute to the occurrence of malaria. Nearly one-third of the children who participated in this study were from families with no formal education, accounting for the highest share of malaria-positive children, while children from educated parents (diploma and above) contribute the least. Children of families with a monthly income of less than 500 ETB top the positive cases found in the study, whereas those from families with a relatively higher monthly income of greater than 2000 ETB contribute the least to the positive share [Table 4]. The net ownership of the insecticides treated recorded in this study, 61.2%, was better compared to that reported by Cameroon, 41.9% [38]. The risk of infection was higher for children whose parents or caregivers had never received any kind of formal education than for those whose parents or caregivers had received education about malaria. Regular use of ITN is considered a successful vector control strategy to stop malaria spread since it may reduce the probability that people will be bitten by mosquitoes [39]. The results of this study also showed that children who did not use ITN regularly had a more than four-fold higher risk of contracting the *Plasmodium* parasite than their counterparts. This study revealed that ITN use has a significant association with malaria [Table 5], which is supported by a study conducted on determinants of malaria among children under the age of five years in Ethiopia by Aychiluhm et al. [40]. This can be because malaria prevention and control strategies vary from region to region. The endemicity of malaria and subsequent measures to control it among a vulnerable population in different areas may be the cause of these variations. Therefore, local authorities in malaria-endemic areas must consider the regular use of ITNs and IRS services, including in the current study area.

### Limitations of the study

This study was carried out using the commonly used microscopy method, which could be the reason why a low prevalence of malaria was recorded. Therefore, the magnitude of the disease could be higher than the one found if high-sensitivity techniques such as polymerase chain reaction and loop-mediated isothermal amplification were used.

## Conclusions and recommendations

Malaria continues to be the greatest public health problem for children aged under five years in the study area. This study revealed a high prevalence of malaria, with children in the age range of 37–59 months taking the largest share. The inability to use ITN, residence, and the presence of vector breeding grounds were among the important associated factors. Therefore, stakeholders (governmental and nongovernmental) organizations should focus on raising the public’s awareness of the use of ITNs, IRS services, and environmental sanitation.

## Supporting information

S1 Data The SPSS dataset. (SAV)

S1 File The English questionnaire. (DOCX)

S2 File The test protocol for malaria microscopy. (DOCX)

S1 Checklist The STROBE statement checklist for cross sectional study. (DOCX)

## Abbreviations

AIDS: Acquired Immune Deficiency Syndrome
ANC: Antenatal Care
AOR: Adjusted Odds Ratio
B/F: Blood Film
COR: Crude Odds Ratio
HIV: Human Immune Virus
IRS: Insecticide Residual Spray
ITN: Insecticide-Treated Net
SOPs: Standard operating procedures
SPSS: Statistical packed for social science
SSA: Sub-Saharan Africa
WBC: White Blood Cell
WHO: World Health Organization
