# Supplementary material for: Malaria prevalence and associated factors among symptomatic children aged under five years attending Sheko District Health Center, Southwest Ethiopia: A cross-sectional study
Source: PLoS One. 2023 Dec 1;18(12):e0295237. doi: 10.1371/journal.pone.0295237 (PMC10691728; doi:10.1371/journal.pone.0295237)
Supplement: S1 File — (DOCX) [file pone.0295237.s002.docx]

# ANNEX-I QUESTIONNAIRE

**MIZAN TEPI UNIVERSITY COLLEGE OF MEDICINE AND HEALTH SCIENCES COLLEGE DEPARTMENT OF MEDICAL LABORATORY SCIENCES.**

This questionnaire is prepared to assess malaria infection and associated factors among symptomatic children under five years of age attended at Sheko District Health Center, Southwest Ethiopia from June to October 2022. So, I invited you to give a piece of valuable information about your child and I also guarantee all information you give me will be kept confidential. Finally, I ask for your cooperation and patience until I finish my question.

Are you voluntary? A. Yes
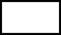
 B. No
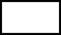


**I. Socio-demographic status**

1. Age of your child **-------------** ID**------------------**

2. Sex A. Male
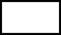
 B. Female
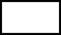


3. Resident A.Urban
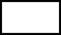
 B.Rural
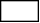


4. Occupational status of the parents/guardians

A. Farmers

B. Housewives

C. Employed

D. Daily laborer

E. Merchant

F. Others

5. Educational status of parents/guardians

1. 1-4 B. 5-8
2. 9-12 D. Diploma and above

6. Marital status of guardians

A. Married
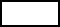
 B. Single
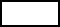
 C. Widowed
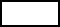
 D. Divorced
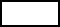


7. Monthly income of guardians /the family

1. < 500 birrs
2. 500-1000 birrs
3. 1001- 2000 birrs
4. > 2000 birrs

8. Family size of guardians

1. < 5 members
2. ≥ 5 members

II. **Assessing associated factors**

1. Is there any mosquito bed net in your house?
2. YES
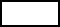
 B. NO
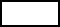

3. If yes, how many bed nets are there in the house?

A. one ITN B. ≥ 2ITNs

1. Did your child sleep in it?

A. Yes
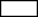
 B. No
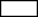


1. If yes how often?
2. Regularly
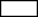
 B. Occasionally
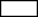
 C. When feeling ill
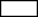
 D. Others
3. Did your house sprayed with insecticide chemical/IRS? A. Yes
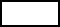
 B. No
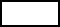

4. If yes how often?
   - - 1. Once per year 2. Twice more per year
5. Did your house have windows? A. Yes
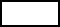
 B. No
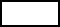

6. If yes does it have a screen? A. Yes
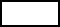
 B. No
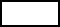

7. Did the wall of your house have a hole in it? A. Yes
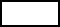
 B. No
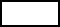

8. Does your house have eaves? A. Yes
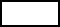
 B. No
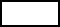

9. Is there a mosquito breeding site around your house area? A. Yes
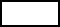
 B. No
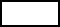

10. If yes, of the following which one?
11. Stagnant water
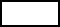
 B. Pond
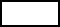
 C. Ditches
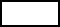
 D. Others(specify)----
12. Do you have a television or radio in your house? Yes
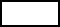
 B. No
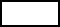

13. Distance from vector breeding site? ----------------------------------
14. Do you think malaria is treatable A. Yes
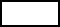
 B. No
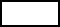

15. Do you think malaria can be prevented/controlled A. Yes
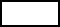
 B. No
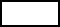

16. Which malaria prevention methods did you know

1. ITN 2. IRS 3. Drugs 4. Environmental mgt

26. Which mode of malaria transmission did you know?

1. Mosquito bite 2. Patient contact 3. Unclean water 4. Bad weather

27. Do you think malaria can be transmitted from person to person? A. Yes
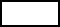
 B. No
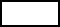


**ANNEX-2 LABORATORY REPORT FORMAT**

**Sheko Health Center**

I.D. ------------------- Date **------------------**

28. **Blood film result**

1. *Plasmodium vivax ------- C. Plasmodium falciparum----*
2. Mixed infections **-------- D.** No Heamoparasites Seen----

29**. Parasite density** (Parasites/µL) **---------------------**
