# Supplementary material for: Malaria prevalence and associated factors among symptomatic children aged under five years attending Sheko District Health Center, Southwest Ethiopia: A cross-sectional study
Source: PLoS One. 2023 Dec 1;18(12):e0295237. doi: 10.1371/journal.pone.0295237 (PMC10691728; doi:10.1371/journal.pone.0295237)
Supplement: S2 File — (DOCX) [file pone.0295237.s003.docx]

**Malaria Microscopy**

**Collection of Finger- Prick Blood and Preparation of Thick and Thin Blood Films**

**Procedure**

- Prepare a glass slide that has been precleaned and the other materials needed for blood collection. Select the 3^rd^ or the 4^th^ finger from the thumb (or big toe for infants). Hold the patient’s hand, palm facing upwards, and clean the selected finger with a 70% ethanol or alcohol swab. Use firm strokes to remove dirt and oils from the ball of the finger and to stimulate blood circulation. Let the alcohol dry from the finger.
- Prick the finger (or big toe) with a new, sterile lancet for every patient. Apply gentle pressure to the finger (or toe) and express the first drop of blood. Wipe the first drop of blood with dry cotton, making sure that no cotton strands remain on the finger that might stick to the blood.
- Make both thick and thin blood films (for one patient) on the frosted side of the same slide.
- To prepare the thin film, place the edge of a clean “spreader” slide at a 45^o^ angle in front of the blood drop intended for the thin film.
- Slowly pull the “spreader” back until it touches the drop of blood and it spreads along the edge of the “spreader”.
- Rapidly push the “spreader” forward (away from the center) in a smooth, continuous motion, until the spreader leaves the bloody part of the slide leaving a “feathery” end for the thin film.
- With the corner of the same “spreader” used for making the thin film, make the thick film by swirling the 3 drops of blood together forming a circle of about 1.2 cm in diameter.

**Staining of malaria blood films**

To prepare staining of malaria blood films with Giemsa, there are three steps to follow: First, prepare the buffered water to a pH of 7.2 for use in the preparation of Giemsa stain solution. Second, prepare the working solution of Giemsa stain for routine staining of malaria blood film. Then, malaria blood films are stained with Giemsa stain.

**Principle**

Freshly-prepared working solution of Giemsa, made from well-prepared stock and diluted with water buffered to pH 7.2 is recommended to achieve optimal staining quality of malaria blood films. Giemsa stock solution prepared for the national program is standardized to minimize the need for frequent adjustment of SOPs for staining.

A properly stained blood film is critical for malaria diagnosis, especially for the precise identification of malaria species. The use of Giemsa stain is the recommended and most reliable procedure for staining thick and thin blood films. Giemsa solution is composed of eosin and methylene blue (azure). The eosin component stains the parasite nucleus red, while the methylene component stains the cytoplasm blue. The thin film is fixed with methanol. Dehemoglobinization of the thick film and staining take place at the same time during the process.

An ideal pH of 7.2 is required to demonstrate the stippling of the parasites to allow proper species identification.

**Examining thick and thin malaria blood films**

In the thick film, the red blood cells (RBCs) are lysed and dehemoglobinized while the malaria parasites are left intact and concentrated allowing their proper detection and identification. In the thin film, when fixed with absolute methanol, the RBCs retain their original morphology, and if malaria parasites are present, become visible inside the cells. Malaria diagnosis must be based on well-prepared thick and thin malaria blood films to ensure correct speciation and accurate estimation of parasite density.

**Detection and identification of the *Plasmodium* species and stages**

- Place the Giemsa-stained blood film to be examined on the microscope stage.
- Position the thick film in line with the objective lens.
- Switch on the microscope and adjust the light source optimally by looking through the ocular and the x10 objective. (low power)
- Place a drop of immersion oil on the thick film and allow it to spread.
- To avoid cross-contamination, ensure that the immersion oil applicator never touches the slide.
- Scan the blood film for parasites and blood elements. Select a part of the film that is well stained and has evenly distributed white blood cells.
- Switch to x100 oil immersion objective over the selected portion of the thick film.
- Raise the mechanical stage until the objective lens gently touches the immersion oil but not the slide.
- Examine the slide systematically. Start at the top left end of the film and begin at the periphery of the field, then move horizontally to the right, field by field.
- Alternatively, from the top left end of the film, move vertically downwards to the next adjacent fields. When the other end of the film is reached, move the slide to the right, then go upwards to the adjacent fields, and so forth.
