# Supplementary material for: Malaria prevalence and associated factors among symptomatic children aged under five years attending Sheko District Health Center, Southwest Ethiopia: A cross-sectional study
Source: PLoS One. 2023 Dec 1;18(12):e0295237. doi: 10.1371/journal.pone.0295237 (PMC10691728; doi:10.1371/journal.pone.0295237)
Supplement: S1 Checklist — (DOCX) [file pone.0295237.s004.docx]

# STROBE Statement—checklist of items that should be included in reports of observational studies

|  | Item No. | Recommendation | Page  No. | Relevant text from the manuscript |
| --- | --- | --- | --- | --- |
| **Title and abstract** | 1 | (*a*) A cross-sectional study design was employed |  |  |
|  |  | (*b*) Capillary blood samples were collected from 286 systematically selected febrile children under 5 years of age. Thin and thick blood smears were prepared, stained with 10% Giemsa working solution, and examined under a light microscope. Data on socio-demographics, associated factors, guardians/caregivers' knowledge, and malaria prevention practices were collected using a pretested structured questionnaire.  Malaria affected 23.4% (95% CI = 18.6–28.8%) of all children under 5 years old who attended the Sheko district health center. *Plasmodium falciparum, Plasmodium vivax*, and mixed infections (both species) accounted for 52.2%, 34.3%, and 13.4% of the cases, respectively. Multivariable logistic regression analysis revealed that malaria infection was significantly associated with children between the ages of 12 and 36 months (AOR = 5.050; 95% CI: 1.964–12.982), children who lived in rural areas (AOR = 2.901; 95% CI: 1.439–5.845), and children who used an insecticide-treated net (ITN) the night before blood sample collection (AOR = 3.341; 95% CI: 1.646–6.781). |  |  |
| Introduction | | | |  |
| Background/rationale | 2 | Malaria is a serious public health issue that continues to cause disease and fatalities. It is a significant contributor to poverty in environments with limited resources, notably in tropical areas around the world. Malaria cases increased from 2019 to 2020, according to data from 85 countries with high malaria prevalence [1].  Infants, children under the age of five, pregnant women, people living with HIV/AIDS, non-immune migrants, mobile populations, and travelers are all at a significantly higher risk of contracting malaria and developing serious illnesses [3, 4]. Beyond the confines of hospitals, malaria's effects can also be felt in people's homes and daily lives. Children who experience severe malaria attacks may experience long-lasting neurological effects, and both mild and severe episodes can cause minor developmental and cognitive problems [5, 6]. Currently, there are challenges in identifying the best ways to maintain high ITN coverage between mass-distribution campaigns, improving case management at the facility and community levels, and overcoming behavioral challenges to encourage proper and consistent use of ITNs and reduce presumptive use of artemisinin combination therapies, both by health workers and community members [2, 7].  There is no sufficient data on the prevalence of malaria among febrile children under 5 years old visiting health facilities, despite the disease remaining among the top 10 diseases in the community. |  |  |
| Objectives | 3 | The current study's objective was to give up-to-date data on the severity of malaria infection among children under five years at the Sheko Health Centre, Southwest Ethiopia, where no data on children under the age of five have been documented. The study also aimed to identify risk factors for *Plasmodium* infection in this community. |  |  |
| Methods | | | |  |
| Study design | 4 | An institutional-based cross-sectional study was conducted in June-October,2022 at Sheko District Health Centre. |  |  |
| Setting | 5 | An institutional-based cross-sectional study was conducted in the Sheko district at Sheko Health Centre, Southwest Ethiopia from June-October,2022. It was established in 1953 E.C. and is located 588 km from Addis Ababa, Ethiopia, and 20 km from Mizan Teferi, the major city of the region. |  |  |
| Participants | 6 | Inclusion and exclusion criteria for participants: Children under the age of five (59 months) who were available during the study period and whose parents or guardians signed an informed written consent form and expressed a desire to participate in the study were eligible. All children under the age of five who received antimalarial medication within 42 days before data collection were excluded.  A systematic random sampling technique was used to select the study participants. |  |  |
| Variables | 7 | Malaria prevalence was the dependent variable whereas, variables were age, sex, residence, educational status, occupational status, family monthly income, bed net (ITN), mosquito/vector breeding site, insecticidal residual spraying (IRS), housing condition, and family size were among the independent variables. |  |  |
| Data sources/ measurement | 8* | **Data sources/measurement**  Face-to-face interviews with parents and guardians of study participants (children) were used to collect socio-demographic information and related factors. A capillary blood sample of 6 μl and 2 μl volume was taken aseptically from the children's fingers or big toes using sterile blood lancets to prepare thick and thin blood film smears.  **Sample processing**  Prelabelled slides were used to make a thin and thick blood smear, and the blood films were air-dried before the thin film was fixed with absolute methanol. Following the standard operating procedure, both thin and thick blood films were stained for 10 minutes with Giemsa stain working solution. The blood film slides were examined by experienced laboratory technicians at the Sheko District Health Centre. |  |  |
| Bias | 9 | To reduce the occurrence of any possible bias systematic random sampling technique was used. |  |  |
| Study size | 10 | The sample size was determined by the following formula.  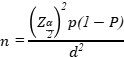  Where n=sample size, Zα/2=1.96, the standard normal variation at 95% confidence level, d=margin of error, 0.05, p=probabilities of the prevalence of disease, 22.8%/0.228 [8], q=probability of failure, which is 1-p=0.772, using the above formula, we have  $\frac{{1.96}^{2}\times0.228\times(1-0.228)}{{0.05}^{2}}$ ≈ 270  Our total population is less than 10, 000 so we use the correction factor as follows  n=n/1+n/N → n=270/1+270/1414 → n ≈ 270  After adding a 10% non-response rate the total sample size was calculated to be 297 and a systematic random sampling technique was used to select **286** study participants/children |  |  |

Continued on the next page

| Quantitative variables | 11 | Quantitative data were entered into the Epi Data Manager (v4.0.2.101) template and exported to Statistical Package for Social Sciences (SPSS) version 25 for analysis. |  |  |
| --- | --- | --- | --- | --- |
| Statistical methods | 12 | Data were entered into Epi Data Manager (v4.0.2.101) and analyzed using Statistical Package for Social Sciences (SPSS) version 25. Descriptive statistics were utilized to summarize the demographic profile of the study participants. To determine the relationship between independent variables and the outcome/dependent variable, both bivariate and multivariable logistic regression analyses were performed. To address missing data, variables were transformed/recoded and replaced with values which stands for negative figures. To control the effects of confounding variables, an adjusted odds ratio was calculated. A P-value less than 0.05 was taken as statistical significance. |  |  |
| Results | | | | |
| Participants | 13* | A total of 286 febrile children under the age of five participated in the study, with a response rate of 96.3%. Of them, 23.4% (95% CI = 18.6–28.8) were confirmed to be positive for *Plasmodium* species. |  |  |
| Descriptive data | 14* | More than 60% of study participants were urban dwellers, while the malaria parasite was more common among children living in rural areas. More than one-third of the study participants were in the age range of 12–59 months, and more than half of them were male. Children between the ages of 37 and 59 months and 12 and 36 months had a higher prevalence of malaria infection than those under the age of 12 months. In comparison to females, males had a greater prevalence of malaria. In terms of *Plasmodium* species distribution, *Plasmodium falciparum* was comparatively more prevalent in both sexes than Plasmodium vivax [Table 1]. |  |  |
| Outcome data | 15* | Malaria affected 23.4% (95% CI = 18.6–28.8%) of all children under 5 years old who attended the Sheko district health center. In terms of parasite density, moderate parasitemia predominated, followed by low and high parasitemia (49.3%, 35.8%, and 14.9% of children, respectively). Males were found to have a larger prevalence of moderate malaria parasitemia than females, making up 57.6% of the total; however, there was no statistically significant difference in parasite density (p =.228). The age range of 37–59 months had the highest parasite density in moderate parasitemia, and there was statistical significance in parasitemia across age ranges (p =.001). |  |  |
| Main results | 16 | Overall, 23.4% (95% CI = 18.6–28.8) of study participants were confirmed to be positive for *Plasmodium* species. The majority of infected children had a moderate parasite density, followed by low and high parasite densities, which accounted for 49.3%, 35.8%, and 14.9% of malaria-positive children, respectively. More than sixty percent of the study participants reported that they had ITN access, and 59.8% of them had used IRS services in the preceding year. The guardians of the children responded that 59.1% of their children used an ITN the night before the blood sample collection, while only 33.6% of them slept under an ITN regularly over the previous two weeks. Binary logistic regression was used to select factors to be fitted to the multivariable logistic regression model, including age (12–36 months), residence, ITN use the night before sample collection, frequency of ITN use, and the presence of vector breeding sites close to the homes of the children. In the multivariable logistic regression analysis, children in the age interval of 12-36 months were found to be more likely to be infected with malaria (AOR = 5.050; 95% CI: 1.964–12.982) than those below 12 months and above three years; similarly, children who were rural residents were more prone to malaria infection than their counterparts (AOR = 2.901; 95% CI: 1.439–5.845). Moreover, children who used ITN the preceding night had a 3.3 times increased chance of contracting malaria than children who did not use it (AOR = 3.341; 95% CI: 1.646–6.781). Furthermore, children who used ITN only occasionally or infrequently had a higher risk of malaria infection than those who used ITN regularly (AOR = 4.375; 95% CI: 1.709-11.203), and children who lived near a vector breeding site had a higher risk of infection (AOR = 33.782; 95% CI: 8.668-131.659) than those who lived far away from a vector breeding site (Table 5). |  |  |

| Discussion | | | | |
| --- | --- | --- | --- | --- |
| Key results | 18 | Overall, 23.4% (95% CI = 18.6–28.8) of study participants were confirmed to be positive for *Plasmodium* species. The majority of infected children had a moderate parasite density, followed by low and high parasite densities, which accounted for 49.3%, 35.8%, and 14.9% of malaria-positive children, respectively. |  |  |
|  |  |  |  |  |
| Limitations | 19 | This study was carried out using the commonly used microscopy method, which could be the reason why a low prevalence of malaria was recorded. Therefore, the magnitude of the disease could be higher than the one found if high-sensitivity techniques such as molecular methods (polymerase chain reaction) and concentration techniques (formol-ether concentration techniques) were used. |  |  |
| Interpretation | 20 | The overall prevalence of malaria was 24.3% (95% CI = 18.6 - 28.8) among children who visited the Sheko district health center. This result was comparable to one obtained in the Arba Minch Zuria District of South Ethiopia (22.1%) [4], the Arsi Negelle Health Centre, Southern Ethiopia [8], a systematic review and meta-analysis; Ethiopia (22.03%) [10], Ziquala district (24.6%) [11], and Nigeria (22.6%) [12]. The prevalence of malaria in this study was higher than the prevalence determined by a study conducted in Borno state, Nigeria (10.3%) [13], Sherkole refugee camp, Ethiopia (3.9%) [14], and Madagascar (7.8 %) [15], sub-Saharan Africa (18.8%) [16] and Uganda (19.5%) [17]. On the contrary, the prevalence determined in this study showed a lower prevalence of malaria than a study conducted in Afar Region Ethiopia (64%) [18], Hadiya Zone Ethiopia (25.8%) [19], Malawi (35.4%) [20], Burkina Faso (49.7%) [21], Ghana (40.5%, 43.0%) [22, 23] and Nigeria (27%) [24]. Although the current study was carried out during the high season for malaria transmission, a low prevalence of malaria parasites was recorded; this may have been the result of geographical variation. This heterogeneity may also be a result of the different regional variations and malaria prevention and control initiatives carried out in the studied areas. *Plasmodium falciparum* and *P. vivax* were the two species found in the blood of children, accounting for 52.3% and 34.3% of infections, respectively. This result is in line with the national Plasmodium species distribution, in Ethiopia [25-27]. *Plasmodium falciparum* showed dominance over *P. vivax,* which can be explained by factors such as the fact that *P. falciparum* has a high multiplication in red blood cells and the study areas have lowland climatic conditions, where *P. falciparum* is a widespread species. In this study, similar to a study conducted in the Wogera district of Ethiopia [28], the number of children who had febrile illness and *Plasmodium* infection decreased with the child's increasing age. This could be because these children live in areas where malaria transmission is stable and can develop age-related protective immunity as a result of continuous exposure to infective mosquito bites [29].  The majority of infected children had a moderate parasite density, followed by low and high parasite densities, which accounted for 49.3%, 35.8%, and 14.9% of malaria-positive children, respectively. This is in line with previous studies carried out in Northern Ethiopia [30] and the contrary study from East Central Tanzania showed a high parasite density [31], while another finding from Ethiopia revealed a significant percentage of low parasite density [32]. Various studies in Ethiopia [14, 28, 30] have shown that malaria vector density and living close to a water body like a river or stream could be important factors influencing malaria transmission. |  |  |
| Generalisability | 21 | The findings of this study could be inferred or generalized to children under five years who attend the health center during the study period. |  |  |
| Other information | |  | | |
| Funding | 22 | No specified fund was received for this study. |  |  |

Continued on next page *Give information separately for cases and controls in case-control studies and, if applicable, for exposed and unexposed groups in cohort and cross-sectional studies.

**Note:** An Explanation and Elaboration article discusses each checklist item and gives methodological background and published examples of transparent reporting. The STROBE checklist is best used in conjunction with this article (freely available on the Web sites of PLoS Medicine at http://www.plosmedicine.org/, Annals of Internal Medicine at http://www.annals.org/, and Epidemiology at http://www.epidem.com/). Information on the STROBE Initiative is available at www.strobe-statement.org.
